# Beyond the nose: hearing status in 70 patients with congenital choanal atresia

**DOI:** 10.1007/s00431-026-06900-y

**Published:** 2026-03-31

**Authors:** M.-S. Yılmaz Topçuoğlu, C. Hornberger, P. J. Schuler, I. Baumann

**Affiliations:** Department of Otorhinolaryngology, Head and Neck Surgery, Medical Faculty Heidelberg, University Hospital Heidelberg, Im Neuenheimer Feld 400, Heidelberg, 69120 Germany

**Keywords:** Choanal atresia, Comorbidity, Conductive hearing loss, Sensorineural hearing loss

## Abstract

The aim was to investigate the hearing status of patients with choanal atresia and to gain new knowledge about the hearing status of patients with choanal atresia. The hearing status of 70 patients with bilateral (*n* = 35) and unilateral (*n* = 35) choanal atresia was evaluated in this cohort study. Demographic data, the prevalence of comorbidities and hearing disorders, as well as the type (conductive vs. sensorineural) and level of hearing loss (HL), and the received type of hearing rehabilitation were investigated. In total, 49% (*n* = 34) of the study cohort had a hearing disorder. Comorbidities were significantly associated to sensorineural (*p* < 0.01) and conductive (*p* < 0.001) HL in the total study cohort. Sensorineural HL was significantly more prevalent in the bilateral (*n* = 15, 43%) than in the unilateral group (*n* = 3, 9%; *p* < 0.01). Conductive HL was observed in the bilateral (*n* = 6; 17%) and unilateral group (*n* = 10; 29%; *p* = 0.394). Rehabilitation consisted of paracentesis/ventilation tube insertion for conductive HL, and hearing aids and cochlear implants for sensorineural HL.

*Conclusion*: Almost every second child in the study cohort presented a hearing disorder. Hearing disorders were correlated with comorbidities. Both, sensorineural and conductive HL were prevalent.This highlights the importance of performing adequate hearing tests and initiating hearing rehabilitation where necessary for patients with bilateral and unilateral choanal atresia. This is crucial for children’s development.

**Table:** 

**What is Known:**
• *Normal hearing is essential for speech, language, and psychosocial development in children.* • *Data on hearing outcomes in patients with choanal atresia are limited.*
**What is New:**
• *Hearing impairment was present in almost half (49%) of a rare cohort of 70 patients with choanal atresia.* •* Sensorineural hearing loss was more prevalent in bilateral choanal atresia and associated comorbidities, whereas conductive hearing loss predominated in unilateral cases.*

• *Normal hearing is essential for speech, language, and psychosocial development in children.*

• *Data on hearing outcomes in patients with choanal atresia are limited.*

• *Hearing impairment was present in almost half (49%) of a rare cohort of 70 patients with choanal atresia.*

•* Sensorineural hearing loss was more prevalent in bilateral choanal atresia and associated comorbidities, whereas conductive hearing loss predominated in unilateral cases.*

## Introduction

Although several studies have discussed the rhinological challenges experienced in patients with choanal atresia (CA), only few have focused specifically on their hearing status [1–3]. Generally, it is well known that children often experience recurrent effusions in the middle ear, resulting in temporary hearing loss (HL) [4]. Also, sensorineural HL is a relevant issue in children [5–7]. However, good hearing is essential for the general and the speech development in patients, and affected children must be monitored closely for hearing issues by a paediatrician and the responsible otorhinolaryngologist [4, 5, 8, 9]. Fortunately, newborn hearing screening has become a standard part of the postnatal examination routine in many countries [10, 11]. HL in children with CA might be associated with syndromic conditions such as CHARGE-syndrome (coloboma, heart defect, atresia choanae, retarded growth and development, genital abnormality, ear abnormality) or trisomy 21 [12–14]. Although clinicians often arrange hearing tests for syndromic patients with CA due to the known link between various syndromes and sensorineural HL, this may be underrepresented in non-syndromic patients with CA, despite the fact that these patients often present with potential risk factors for congenital hearing disorders, such as prematurity or neonatal intensive care unit treatment [6, 15, 16]. It therefore stands to reason that patients with CA should undergo audiological assessment, independent from the presence of a comorbidity. Urbancic et al. and Moreddu et al. recommended hearing tests for patients with CA [17, 18]. Nevertheless, in our experience, preoperative hearing tests are not carried out routinely in the context of planned surgical CA repair [19]. As research into the hearing status of patients with CA is still insufficient, this study is the first to examine a rare cohort of 70 CA patients. The main aim of the study was to determine the need for standardised perioperative hearing diagnostics and to investigate the prevalence, types and levels of hearing disorders in patients with CA.

## Materials and methods

### Patients, study design, and ethics

The hearing test results of 70 patients with congenital CA, who were diagnosed and treated in the Otorhinolaryngology Department of a German university hospital between January 2010 and October 2025, were retrospectively screened. The patients underwent age-appropriate hearing tests, such as objective tympanometry, otoacoustic emissions and brainstem evoked response audiometry, as well as subjective pure tone audiometry as a behavioural response measurement, and the localisation of children’s songs in a free field. The study was approved by the local ethics committee (S-511/2022). Adherence to all ethical standards as per the current revision of the Declaration of Helsinki of 2024 was ensured.

### Assessed data

The primary endpoints were the prevalence and type (sensorineural versus conductive), and, in cases of sensorineural HL, also the level of HL according to the World Health Organization’s classification (Table 1) [7]. In addition, existing conductive HL due to tympanic effusion, defined by flat tympanometries, was defined as a further category of hearing impairment. Imaging as computed tomography (CT) or magnetic resonance imaging, if available, was examined to identify any inner ear malformations. The type of hearing rehabilitation was recorded. The presence of comorbidities was categorised in no comorbidity, a non-syndromic comorbidity (e.g. oesophageal atresia, epilepsy, non-syndromic-related heart defects) and syndromic comorbidities (e.g. CHARGE-syndrome or trisomy 21). Genetic testing conducted by the attending paediatricians for all patients with bilateral CA has enabled the diagnosis of syndromic comorbidities. Patients with unilateral CA only underwent genetic testing if further comorbidities were suspected. Age at the time of hearing test, age at the time of surgery, gender, affected side(s) of CA, and concomitant surgical procedures as adenoidectomies, paracentesis and ventilation tube insertions were collected.

**Table 1:** Levels of sensorineural hearing loss according to the World Health Organisation [7]

Levels of hearing loss	Hearing Threshold* [dB]
Normal hearing	< 20 dB
Mild	20 to < 35 dB
Moderate	35 to < 50 dB
Moderately severe	50 to < 65 dB
Severe	65 to < 80 dB
Profound	80 to < 95 dB
Complete/deafness	> 95 dB

*Threshold relates to the better hearing ear in decibels [dB]

### Statistical analysis

The data were analysed using GraphPad Prism version 10.4.0 (GraphPad Software, Boston, Massachusetts, USA). The age at surgery in months was presented as median, range, and interquartile range (IQR). The Mann–Whitney-*U* test was applied to test for age differences between the groups. Further descriptive data on affected sides of CA, gender distribution, and presence of comorbidities were given as absolute and relative numbers. The Fisher’s exact test was utilized for the inter- and intra-group comparisons of the prevalence of comorbidities and HL, and their association. A *p*-value of *p* < 0.05 was considered statistically significant.

## Results

### Age-appropriate hearing tests were available in 70 patients with congenital choanal atresia

In total, 70 patients with CA were included (Table 2). 35 had bilateral CA (20 females, 15 males), and 35 patients had unilateral CA (23 × right-sided, 12 × left-sided; 23 females, 12 males; Table 2). Patients with bilateral CA were significantly younger at time of surgery with a median age of 3 months (range 0–368 months; IQR 18 months) compared to the patients with unilateral CA with a median age of 33 months (range 2–212 months, IQR 60 months; *p* < 0.001). This difference also applied to the age at the time of the hearing test (*p* < 0.01; Table 2).

**Table 2:** Demographics

Variable	Bilateral	Unilateral	*p*-values*
Patient numbers	35 (100)	35 (100)	
Age at hearing test [months]	6; 0–180; 17*	28; 7–90; 55	< 0.01
Age at surgery [months]	3; 0–368; 18*	33; 2–212; 60	< 0.001
Gender *n* (%)			
Female	20 (57)	23 (66)	
Male	15 (43)	12 (34)	
Side *n* (%)			
Right	-	23 (66)	
Left	-	12 (34)	
Comorbidities *n* (%)	27 (77)*	11 (31)	< 0.001
Non-syndromic	9 (26)	4 (11)	
Syndromic	18 (51)*^#^	7 (20)	< 0.05
CHARGE	8 (23)	4 (11)	
None	8 (23)	24 (69)	

70 patients with choanal atresia could be included. Age at the time of hearing test and at the time of surgery is given as median; range; interquartile range. All other variables are given as absolute number (relative number). *significant difference in the comparison of bilateral to unilateral group in the same variable (*p* < 0.05). #significant difference in the intra-group comparison of the bilaterally affected patients between the prevalence of non-syndromic and syndromic comorbidities (*p* < 0.05). Non-syndromic comorbidities included heart defects, stunted growth, oesophageal atresia, epilepsy, anal atresia. Syndromic comorbidities included Axenfeld-Rieger-Syndrome, CHARGE-Syndrome, Deletionsyndrome, Pfeiffer-Syndrome, Treacher-Collins-Syndrome, Trisomy 21, Williams-Beuren-Duplication-Syndrome. As the CHARGE- syndrome was observed in the patient cohort, and is often associated with HL, the sole number of patients with CHARGE-syndrome is additionally displayed

### Sensorineural hearing loss is more prevalent in patients with bilateral choanal atresia

49% (*n* = 34/70) of the total study cohort had a hearing disorder. Of the hearing disorders, 26% (18/70) were sensorineural, and 23% (16/70) were conductive.

**Fig. 1:**
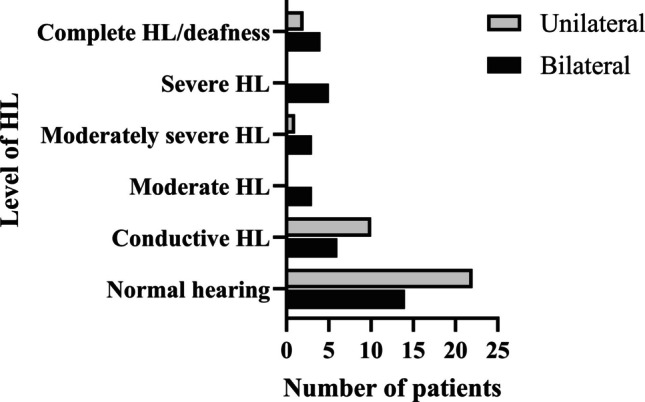
Levels of hearing loss in the patient cohort, classified according to [7]. Two patients with unilateral choanal atresia and CHARGE-syndrome presented with single-sided deafness and moderate to severe HL in their other ear. This figure included their worst ears, meaning both patients were accounted for in terms of complete hearing loss/deafness. *HL* Hearing loss

In the bilateral group, a total of 21 patients (60%) were found to have a hearing disorder (Fig. 1). Of these patients, 15 (43%) had sensorineural HL, while six (17%) had conductive HL. All patients of the bilateral group presented with symmetric HL.

In the unilateral group, a total of 13 patients (37%) had a hearing disorder (Fig. 1). Of these patients, three (9%) had sensorineural HL, while ten (29%) had conductive HL. Two patients with CHARGE-syndrome presented with single-sided deafness and moderate to severe HL in their other ear. All other patients presented with symmetric HL.

The overall prevalence of hearing disorders, namely sensorineural HL and conductive HL, did not differ significantly between the bilateral and unilateral group (*n* = 21/35, 60% vs. *n* = 13/35, 37%; *p* = 0.094). Also, the prevalence of the different levels of sensorineural HL (Table 1, Fig. 1) did not differ between the bilateral and the unilateral group (*p* = 0.054–0.673). However, the overall prevalence of sensorineural HL was significantly higher in the bilateral group than in the unilateral group (*p* < 0.01). Conductive HL was not more prevalent in the bilateral group (*n* = 6/35, 17%) compared to the unilateral group (*n* = 10/35, 29%, *p* = 0.394).

For the intra-group comparisons in the bilateral group, the sensorineural HL (*n* = 15/35, 43%; *p* > 0.999) and the conductive HL (*n* = 6/35, 17%; *p* = 0.063) were not more prevalent than normal hearing (*n* = 14/35, 40%). But the sensorineural HL (*n* = 15/35, 43%) was significantly more prevalent than the conductive HL (*n* = 6/35, 15%; *p* < 0.05).

On contrary, for the intra-group comparison in the unilateral group, normal hearing (*n* = 22/35, 63%) was significantly more prevalent than sensorineural HL (*n* = 3/35, 9%; *p* < 0.001) and conductive HL (*n* = 10/35 (29%); *p* < 0.01). The comparison of the prevalence of the conductive HL (*n* = 10/35, 29%) to the prevalence of sensorineural HL (*n* = 3/35, 9%) did not show any significance (*p* = 0.062).

### Comorbidities correlate with conductive and sensorineural hearing loss and are more prevalent in bilateral choanal atresia

**Fig. 2:**
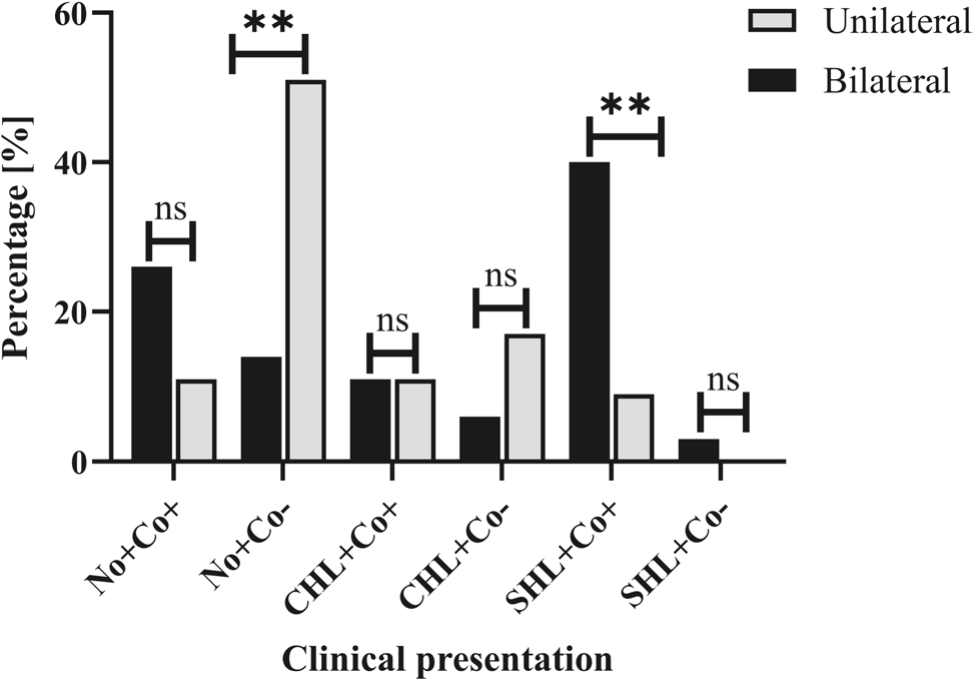
Clinical presentations of hearing and comorbidities. The presence of the hearing status in combination with comorbidities in the total study cohort of *n* = 70 patients is shown as percentage. No: normal hearing (+ yes; − no); Co: comorbidity (+ yes; − no); *CHL* conductive hearing loss (+ yes; − no), *SHL* sensorineural hearing loss (+ yes; − no), *ns* not significant (*p* = 0.218 to > 0.999); **p < 0.01

Comorbidities in general, namely non-syndromic and syndromic comorbidities, were present in 54% (*n* = 38/70) of the total study cohort (Table 2). The general presence of comorbidities (38/70, 54%) in the total cohort was associated with a higher prevalence of both sensorineural (18/70, 26%; *p* < 0.01) and conductive (16/70, 23%; *p* < 0.001) HL. This was also confirmed in the subgroup analysis. Comorbidities in general (bilateral: 27/35, 77%, unilateral: 11/35, 31%) were associated with a significantly higher prevalence of sensorineural HL in both groups (bilateral: 15/35, 43%, *p* < 0.01, unilateral: 3/35, 9%, *p* < 0.05) and with a significantly higher prevalence of conductive HL in the bilateral group (bilateral: 6/35, 17%, *p* < 0.001, unilateral: 10/35, 29%, *p* > 0.999). The unilateral group was significantly more likely to have normal hearing and no comorbidities than those of the bilateral group (*p* < 0.01; Fig. 2). Conversely, the bilateral group had significantly higher rates of sensorineural HL and comorbidity than the unilateral group (*p* < 0.01; Fig. 2).

In the bilateral group, comorbidities in general were significantly more prevalent (*n* = 27; 77%) compared to the unilateral group (*n* = 11; 31%; *p* < 0.001; Table 2). Also, syndromic comorbidities were more prevalent in the bilateral group (*n* = 18; 51%) compared to the unilateral group (*n* = 7; 20%; *p* < 0.05). In the within-group comparison of the bilateral group, syndromic comorbidities (*n* = 18; 51%) were significantly more frequent than non-syndromic comorbidities (*n* = 9; 26%; *p* < 0.05). This did not apply to the unilateral group (Table 2).

### Non-surgical and surgical hearing rehabilitation is available and imaging shows cochlear malformations in cases of sensorineural hearing losses

For conductive HL caused by middle ear effusions, paracentesis and/or ventilation tube insertion without (*n* = 8) and with adenoidectomy (*n* = 8) were performed concomitantly with surgical CA repair in both groups. Patients with adenoidectomy were in median 26 months (range 8–70 months; IQR 37.8). No clear trend towards a more affected side was observed in patients with unilateral CA and conductive HL due to tympanic effusion.

Two patients with CHARGE-syndrome with moderate to severe sensorineural HL, who were fitted with behind-the-ear hearing aids, revealed dysplastic semicircular canals in the performed CT scans, and a mildly dysplastic cochlea in one of them.

The six cases of complete HL, all patients with CHARGE-syndrome, had cochlear implantation for hearing rehabilitation where applicable. In the bilateral group, one patient received cochlear implants in both ears. Imaging revealed a bilateral Mondini malformation in this patient. Three patients of the bilateral group received cochlear implants in one ear due to nerve aplasia in the other ear. Next to the nerve aplasia, imaging revealed a bilateral Mondini malformation in one of these patients, and in the other two patients, moderate dysplastic cochleae and semicircular canals. CT scans described mildly deformed but fully functional ossicles in three of these patients from the bilateral group. In the unilateral group, two patients had asymmetric HL, and received a cochlear implant on one side due to complete HL, and a behind-the-ear hearing aid on the other side due to mild and moderately severe HL, respectively. Imaging revealed bilaterally dysplastic semicircular canals in both patients. Middle ear malformations were not described in their CT scans. One of the patients presented with Mondini dysplasia in both cochleae, while the other patient had mild dysplasia in both cochleae.

## Discussion

Previous publications emphasised the importance of identifying HL in patients with CA [1–3]. Patients with CA can experience chronic ventilation problems of the middle ear [1]. For this reason, Brihaye et al. have reported performing tympanometry on their CA patients, but have not published the results of these tests [20]. Urbancic et al. recommended hearing tests for patients with CA [17]. For patients with bilateral CA, this can be performed postoperatively to address the urgent need for successful airway management [17]. In the consensus paper by Moreddu et al., 57% of authors voted in favour of preoperative audiological testing in patients with CA [18]. Otological care has already been recommended for patients with CHARGE-syndrome due to the high prevalence of middle ear disorders [21]. However, to date, published studies that have examined the hearing status of a large cohort of patients with CA are scarce.

Enhanced hearing tests are recommended for certain high-risk cases, such as premature birth or a postnatal stay in an intensive care unit with invasive ventilation [6, 16]. Given the additional likelihood of an associated comorbidity with potential hearing disorders [22], it is important to have a reliable and fixed workflow for otologic diagnostics and rehabilitation for all patients with CA. While earlier reports on hearing disorders in patients with CA from 1979 and 1986 presented with case series of four, nine and seventeen patients [1–3], the strength of our study was the rare large cohort of 70 patients with congenital CA with documented hearing status. Thus, we could divide our cohort in two equally sized subgroups of bilateral and unilateral CA. We considered this to be important to avoid introducing bias due to the expected prevalence of comorbidities and the potential for associated hearing disorders to be higher in the bilateral group [22–24]. This was confirmed in our study cohort: the prevalence of comorbidities was shown to be significantly higher in the bilateral group than in the unilateral group. All included patients with complete HL and CHARGE-syndrome presented with cochlear malformations. This once again confirms the importance of considering ear malformations in these syndromic patients, including those that are unilaterally affected. However, the importance of monitoring the hearing does not only apply to patients with syndromic CA. We could show, that nearly every second patient of our total study cohort (49%) had a conductive or sensorineural hearing disorder. Compared to the general population of newborns, in which 1–3 out of every 1000 newborns suffer from sensorineural HL [5–7], the prevalence of sensorineural HL in the present total cohort was higher with 26%. This demonstrates that hearing tests often reveal disorders in this patient group and that hearing diagnostics are crucial for determining the most appropriate rehabilitation measures. This is of course particularly of importance if there is an additional comorbidity as this study’s correlation analysis demonstrated that the general presence of comorbidities (including both, non-syndromic and syndromic comorbidities) was significantly associated with sensorineural HL in the total study cohort, as well as in both groups when investigated separately. But, also, in the unilateral group, sensorineural HL was prevalent with 9%.

The bilateral group had a significant higher prevalence of sensorineural HL than the unilateral group. Fortunately, sensorineural HL due to CA remains stable and can be rehabilitated with the help of appropriate hearing aids [21], as conventional behind-the-ear hearing aids, bone conduction hearing aids or cochlear implants. Additionally, our results presented, that also conductive HL is present in bilateral (17%) and unilateral (29%) CA. Conductive HL due to ventilation issues can be alleviated through surgical procedures such as paracentesis, ventilation tube insertion and adenoidectomy, if conservative treatment measures prove ineffective. Contrary to what one might expect due to the narrower anatomy on the CA-affected side, our data did not show that the ear from the CA-affected side in the unilateral group had conductive HL more frequently and the data demonstrated that conductive HL in the unilateral group was not associated with the presence of comorbidities. Rather, these children had bilateral conductive HL. This could be because conductive HL is generally more prevalent in infants, as infant-typical tympanic effusions might also play a role at that age in this patient cohort and the median age at hearing test in this group was 28 months. This is underlined by the median age of 26 months of the patients who needed adenoidectomies due to ventilation issues, so they were no newborns anymore, but infants. This demonstrates the importance of discussing the potential presence of adenoids and their removal as part of the surgical procedure preparation. On the one hand, nasal breathing is facilitated in addition to the opening of the CA. On the other hand, it prevents possible ventilation disorders of the ear. In addition, it is important to emphasise, that comprehensive and repeated audiological evaluation remains essential for these patients, even after Eustachian tube dysfunction has been treated. Given the potential for additional conductive or undetected sensorineural HL in this patient group, objective hearing assessments such as brainstem evoked response audiometry should also be a consistent component of the diagnostic workflow.

The study had some limitations. Firstly, the current World Health Organisation classification of sensorineural HL levels is actually only intended for adults [7]. Nevertheless, we used this classification for the present study cohort due to its clear structure. However, this has no influence on the reported prevalence rates, as the most important distinction was between sensorineural and conductive HL rather than the single sublevels of sensorineural HL (Table 1). Furthermore, it should be emphasised that this study has the strength of an exceptionally large cohort of 70 patients with CA and documented hearing tests, which means that the prevalence within this study cohort can be determined with certainty. Nevertheless, the results cannot be applied to the entire CA patient population without concern. This would require national and international studies and an international CA register. Such a register does not currently exist. The general hearing status of newborns in Germany was discussed above using newborn hearing screening in order to provide comparable data sets. However, as the clinical presentation of CA can range from multimorbidity to isolated CA only, it is not feasible to compare this group with other patient groups or even a healthy group. We divided the cohort into bilateral and unilateral subgroups for comparison. Unfortunately, our study does not allow us to investigate potential biases relating to prolonged neonatal intensive care unit stays, prematurity and HL, as many patients were referred to our ENT department for surgical repair only and received perinatal healthcare elsewhere. A future study could overcome this limitation by using a multicentre, interdisciplinary study design.

Considering that the median age at presentation was 3 months for bilateral CA and 33 months for unilateral CA, it should be noted that dedicated testing for conductive and sensorineural HL, and identification of mixed forms, is very difficult, if not impossible, at this age. In the few cases where the ossicles were radiologically described as mildly dysplastic but functional, more specialised testing at an older age may reveal mixed forms. Unfortunately, this could not be determined due to the young age of the patient cohort, but it should be the focus of future studies. It is noteworthy that the median age at surgery in the bilateral group was lower than the median age at hearing assessment. This discrepancy can be explained by the fact that most children underwent newborn hearing screening shortly after birth as part of the German newborn screening programme. However, many patients in the bilateral group presented with severe syndromic comorbidities (e.g. cardiac malformations or oesophageal atresia), which required subsequent surgeries and prolonged stays in intensive care units. Consequently, a comprehensive audiological assessment in the paediatric audiology department was delayed until the patients were stable enough to undergo it.

Lastly, imaging of the inner ear was not available for all patients, but only for those with complete HL/deafness and two with severe HL. This made it difficult to examine specific rates of middle ear and inner ear malformations in our cohort in more detail. This is why we only provided a descriptive overview of the presented inner ear malformations.

In conclusion, the study’s main focus was to answer the question of whether hearing disorders are prevalent and hearing tests should be performed routinely in all patients with CA. In the investigated study cohort nearly every second child with congenital CA presented with a sensorineural or conductive HL. Consequently, in the authors’ eyes, early and comprehensive hearing tests should be a consistent part of the diagnostic workflow for all patients with CA. They are crucial to provide reliable hearing diagnostics, timely identification, management and rehabilitation of clinically relevant hearing disorders, with the aim of achieving the best possible compensation for their disabilities.

## Data Availability

All data generated or analysed during this study are included in this published article.

## Abbreviations

CA: Choanal atresia
CHARGE: Coloboma, heart defect, atresia choanae, retarded growth and development, genital abnormality, ear abnormality
CT: Computed tomography
HL: Hearing loss
IQR: Interquartile range
